# Detection of *Streptococcus uberis* in Bovine Milk Using a Simplified DNA Preparation Method and Colorimetric LAMP Assay

**DOI:** 10.3390/ani16132029

**Published:** 2026-07-02

**Authors:** Tewodros Fentahun Jember, Mark Edward Westman, Sameer Dinkar Pant, Seyed Ali Ghorashi

**Affiliations:** 1 School of Agricultural, Environmental and Veterinary Sciences, Faculty of Science and Health, Charles Sturt University, Wagga Wagga, NSW 2678, Australia; tjember@csu.edu.au (T.F.J.); spant@csu.edu.au (S.D.P.); 2 College of Veterinary Medicine and Animal Sciences, University of Gondar, Gondar P.O. Box 196, Ethiopia; 3 Elizabeth Macarthur Agricultural Institute (EMAI), Department of Primary Industries and Regional Development (DPIRD), Menangle, NSW 2568, Australia; mark.westman@dpird.nsw.gov.au; 4 Gulbali Institute, Charles Sturt University, Boorooma Street, Wagga Wagga, NSW 2678, Australia

**Keywords:** dairy cattle, *Streptococcus uberis*, mastitis, DNA preparation, colorimetric LAMP, veterinary diagnostics

## Abstract

Bovine mastitis caused by *Streptococcus uberis* (*S. uberis*) remains a major challenge of dairy production system worldwide. This study aimed to develop a colorimetric loop-mediated isothermal amplification (LAMP) assay and a conventional PCR assay targeting the 16S rRNA gene for detection of *S. uberis* in bovine milk. Both assays were evaluated with a simplified HotSHOT (HS) DNA preparation method that can be completed in under 10 min and was compared with a commercial DNA extraction kit before LAMP and PCR testing. LAMP reactions using HS-extracted DNA required an extended incubation time of 30 min, compared with the standard 60 min, to account for the crude DNA preparation. Using tenfold serial dilutions of purified DNA in nuclease-free water, the limit of detection was 1.84 × 10^−4^ ng/µL for LAMP and 1.84 × 10^−5^ ng/µL for PCR. In pasteurised milk spiked with *S. uberis* DNA and extracted by HS, the limit of detection was 10 ng/mL for LAMP and 1 ng/mL for PCR. Using the commercial kit, both methods achieved 1 ng/mL. Among 17 culture-confirmed clinical milk samples extracted by HS, LAMP detected *S. uberis* in 14 samples (82.35%; 95% CI: 0.56 to 0.96), while PCR detected 15 samples (88.24%; 95% CI: 0.64 to 0.99), with no significant difference between methods (McNemar’s test, *p* > 0.05). Both LAMP and PCR showed 100% analytical specificity against 10 non-target bacterial species under the conditions tested. These findings support HS combined with colorimetric LAMP as a practical diagnostic workflow that warrants further validation under field conditions.

## Simple Summary

*Streptococcus uberis* (*S. uberis*) is an important bacterial cause of mastitis in dairy cows. This study developed and evaluated two molecular diagnostic methods, colorimetric LAMP and conventional PCR, for detecting *S. uberis* in milk samples. A rapid and simple Hot Sodium Hydroxide and Tris-HCl (HotSHOT) DNA preparation method was used and compared with a commercial DNA extraction kit. Both tests were able to accurately detect *S. uberis*, with PCR showing slightly higher sensitivity. The assays were highly specific and did not detect other bacterial species. Overall, the combination of the HotSHOT DNA preparation method and the colorimetric LAMP assay offers a rapid, simple, and cost-effective approach for detecting *S. uberis* in bovine milk, with potential application for on-farm and field-based diagnosis. Further validation under field conditions is recommended.

## 1. Introduction

Bovine mastitis is a persistent challenge in dairy production systems worldwide. It results in decreased milk yield, compromised animal welfare and increased use of antimicrobial agents [1,2,3,4]. Bacterial culture remains the standard approach for identifying mastitis pathogens. However, it is labour intensive and typically requires sample transport to centralised diagnostic laboratories [3,5]. Consequently, treatment decisions are often made before the causative agent has been identified [6,7].

Rapid cow-side tests such as the California Mastitis Test and somatic cell count are commonly used methods for mastitis screening without identifying specific bacterial pathogens [8,9]. This absence of pathogen level identification can lead to broad-spectrum antimicrobial treatments that may not be appropriate for the causative microorganism [10,11].

Molecular diagnostic methods enable sensitive and specific identification of mastitis pathogens directly from milk samples. This is because such tests directly detect bacterial nucleic acids, enabling identification of very low bacterial counts and providing high specificity by targeting unique gene sequences of specific bacteria directly, rather than relying on bacterial growth or biochemical analysis [12,13,14]. Conventional polymerase chain reaction (PCR) is widely used for this purpose. However, its dependence on thermal cycling equipment, stable power supply and trained operators restricts its application outside laboratory environments [15,16,17,18]. These limitations and its high cost may reduce the feasibility of PCR as a routine field or farm diagnostic tool [19,20,21,22].

Loop-mediated isothermal amplification (LAMP) has gained attention as a viable alternative to PCR for decentralised testing [23]. LAMP amplifies DNA under constant temperature conditions and does not require sophisticated instrumentation [23,24]. The development of colorimetric LAMP assays has further simplified detection by enabling visual interpretation of results through a colour change, eliminating the need for gel electrophoresis, fluorescence readers or turbidity meters [25]. These characteristics make LAMP well suited for rapid testing in settings where laboratory infrastructure is unavailable [26,27].

Despite the potential of molecular assays, their diagnostic performance depends substantially on the quality of template DNA [28,29,30]. DNA extraction is often the most critical and limiting step in molecular workflows [31]. Rapid DNA extraction methods, while attractive for on-site testing, tend to produce crude preparations that may contain amplification inhibitors [32]. Conversely, commercial DNA extraction kits yield higher quality DNA but involve equipment (heating blocks and centrifuges), multiple reagents and higher costs [33]. This gap between simplified amplification methods and complex extraction procedures remains a barrier to adopting molecular diagnostics outside the laboratory [34].

*Streptococcus uberis* (*S. uberis*) is among the most commonly isolated pathogens from bovine mastitis cases globally [35]. It is primarily associated with environmental transmission, often linked to contaminated bedding materials, soil and pasture [35]. *S. uberis* infections can present as both clinical and subclinical mastitis, and their management may differ from that of contagious pathogens. This makes accurate and timely identification critical for appropriate treatment and herd management [35].

Several LAMP assays have previously been developed for detecting *S. uberis* in milk [36,37]. However, these assays have relied on non-colorimetric detection formats or laboratory-based DNA extraction methods, requiring instrumentation that limits their use in field settings. Combining a colorimetric LAMP assay with a simplified DNA preparation method would address this limitation and support the development of field compatible diagnostic workflows. The HotSHOT (Hot Sodium Hydroxide and Tris-HCl) method is a chemical-based DNA preparation approach that uses alkaline lysis followed by acid neutralisation. It requires only two reagent solutions and basic heating equipment, and can be completed in approximately 10 min, making it suitable for resource-constrained environments and adaptable for on-farm molecular diagnostics. This study aimed to develop and evaluate a colorimetric LAMP assay for the detection of *S. uberis* in bovine milk and to assess its compatibility with a simplified HotSHOT (HS) DNA preparation method. We optimised HS as a chemical-based DNA extraction method from milk samples, suitable for resource-constrained environments and adaptable for on-farm molecular diagnostics.

Pasteurised bovine milk spiked with purified *S. uberis* genomic DNA was used for analytical evaluation. The performance of the HS method was compared with a commercial DNA extraction kit, and clinical bovine milk samples were used to assess diagnostic sensitivity and specificity.

## 2. Materials and Methods

### 2.1. DNA Extraction

The optimised HS DNA preparation method consisted of a lysis solution containing 0.2 mM Ethylenediaminetetraacetic acid disodium salt dihydrate AR (disodium EDTA) (Chem-Supply Pty Ltd., Adelaide, Australia, Cat. No. EA023-100G) and 25 mM Sodium hydroxide (NaOH) (Chem-Supply Pty Ltd., Adelaide, Australia, Cat. No. SL178) adjusted to pH 12.0, and a neutralisation solution containing 40 mM Tris hydrochloride (Tris-HCl) (Merck KGaA, Darmstadt, Germany, Cat. No. H5123) adjusted to pH 1.9. A volume of 150 µL of spiked milk sample was mixed with 75 µL of a lysis solution in a 1.5 mL microfuge tube. First, the mixture was thoroughly mixed by pipetting and gently mixed manually by finger flicking the tube. Second, the mixture was heated at 90 °C for 10 min on a heat block. Third, 75 µL of the neutraliser solution was added and mixed thoroughly by pipetting and gently mixing again by finger flicking the tube yielding a final volume of 300 µL. No centrifugation was required. The detailed HS protocol has been described previously [29].

The Wizard^®^ Genomic DNA Purification Kit (Promega Corporation, Alexandria, NSW, Australia, Cat. No. A1120) was used as the reference commercial extraction method. Extraction was performed according to the manufacturer’s instructions for Gram-positive bacteria. Pasteurised milk spiked with nuclease free water was used as a negative extraction control for both methods.

### 2.2. LAMP and PCR Primer Design

LAMP primers were designed to detect *S. uberis* by targeting the 16S rRNA gene. The target sequence was obtained from the NCBI GenBank database under accession number AB023576.1. Primer design was performed using the Primer Explorer version 5 software (http://primerexplorer.jp/e/index.html; Eiken Chemical Co., Tokyo, Japan; accessed on 13 April 2024).

**Figure 1:**
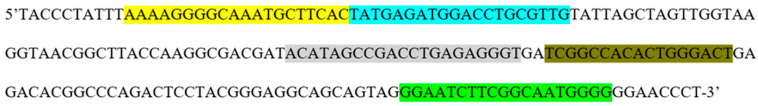
Schematic representation of LAMP primer-binding sites within the 16S rRNA gene of *S. uberis* (GenBank accession AB023576.1). Each primer is colour-coded to indicate its specific binding region. The locations of outer primers (SU-F3 and SU-B3) and the loop primer (SU-LB) are shown with nucleotide coordinates relative to the reference sequence. Inner primers SU-FIP and SU-BIP are composite primers comprising two target specific regions and are therefore not represented by single continuous binding positions.

The LAMP primer set consisted of an outer forward primer (SU-F3), an outer backward primer (SU-B3), a forward inner primer (SU-FIP), a backward inner primer (SU-BIP) and a backward loop primer (SU-LB). Primer-binding sites were mapped relative to the 16S rRNA gene sequence of *S. uberis* (GenBank accession AB023576.1). The SU-F3 and SU-B3 primers were also used as forward and reverse primers for conventional PCR amplification, generating an expected amplicon size of 178 base pairs. All primers were synthesised by Sigma Aldrich Pty Ltd. in Australia. Primer sequences are listed in Table 1, and primer-binding locations within the target sequence are illustrated in Figure 1.

### 2.3. Colorimetric LAMP Assay

The colorimetric LAMP assay was initially developed and optimised using purified *S. uberis* genomic DNA extracted from the same bacterial isolate described in Section 2.1 using the Wizard SV Genomic DNA Purification System (Promega). The assay was performed using the WarmStart^®^ Colorimetric LAMP 2× Master Mix (New England Biolabs, Ipswich, MA, USA, Cat. No. M1800). Each reaction was carried out in a final volume of 20 µL containing 10 µL of 2× LAMP master mix, primers at final concentrations of 0.2 µM each for SU-F3 and SU-B3, 0.4 µM for SU-LB, and 1.6 µM each for SU-FIP and SU-BIP, 5 µL of nuclease free water and 2 µL of extracted DNA.

For negative controls, both distilled water and DNA extracted from non-spiked pasteurised milk were used. For positive controls, 2 µL of *S. uberis* genomic DNA at 1 ng/µL, extracted using the same commercial kit, was included.

LAMP reactions were incubated at 65 °C on a heat block for 60 min. For DNA extracted using the optimised HS method, the LAMP incubation time was extended by an additional 30 min (90 min in total). This adjustment was made to allow sufficient amplification from a crude DNA preparation that may contain lower DNA concentrations or residual inhibitors compared to purified kit-based extractions. This extended incubation time was applied consistently for all experiments using HS-extracted DNA. According to the manufacturer’s instructions, a colour change from pink to yellow was interpreted as a positive result, while no colour change was considered negative. Samples showing intermediate or orange coloration were considered ambiguous and re-tested. Tube interpretation was performed independently by two blinded readers to minimise subjective bias, and discrepant results were resolved by repeat testing and consensus.

### 2.4. Conventional PCR Assay

Conventional PCR was performed using a Rotor Gene^TM^ 6000 thermal cycler (Qiagen, Melbourne, Australia). Each reaction was carried out in a final volume of 25 µL containing 5.0 µL of 5× GoTaq Green Flexi Reaction Buffer, 1.5 µL of MgCl_2_ (25 mM), 2.0 µL of dNTP mix (1250 µM), 0.3 µL of Taq DNA polymerase (5 U/µL; Promega, Australia), SU-F3 and SU-B3 primers (2 µM) and 2.0 µL of extracted DNA. Nuclease-free water was added to reach the final volume. Negative and positive controls were prepared as described for the colorimetric LAMP assay.

PCR amplification was performed with an initial denaturation at 95 °C for 2 min, followed by 30 cycles of denaturation at 95 °C for 20 s, annealing at 58 °C for 30 s and extension at 72 °C for 45 s, with a final extension at 72 °C for 5 min. PCR products (10 µL) were analysed by agarose gel electrophoresis on a 1.8% agarose gel stained with GelRed and visualised using a Gel Doc XR+ imaging system (Bio-Rad, Hercules, CA, USA, Cat No. 41003).

### 2.5. Limit of Detection

The LOD of the LAMP and PCR assays was first assessed using serially diluted purified *S. uberis* genomic DNA. Purified DNA at a stock concentration of 18.4 ng/µL was serially diluted tenfold in nuclease-free water. Each dilution was tested by both LAMP and PCR in triplicate to establish the baseline detection capability of each assay independent of the milk matrix and extraction procedure.

For the spiked milk experiments, the LOD of each DNA extraction method was compared based on the LOD for both colorimetric LAMP and PCR. For each serial dilution, the DNA concentration was expressed as ng/mL and as approximate genome equivalents per mL, based on the estimated genome size of *S. uberis* (approximately 1.8 Mb, corresponding to approximately 2 fg per genome copy). The number of DNA copies entering each reaction was calculated based on the volume of milk used for extraction (150 µL), the elution volume (100 µL for the Wizard^®^ Kit; 300 µL for the HS method) and the template volume added to each reaction (2 µL). For the Wizard^®^ Kit, each reaction contained DNA equivalent to 3 µL of the original milk sample. For the HS method, each reaction contained DNA equivalent to 1 µL of the original milk sample. The LOD was defined as the lowest DNA concentration at which a positive result was consistently obtained across all three replicates for a given assay and DNA extraction method.

### 2.6. Preparation of DNA-Spiked Milk Samples

Commercial pasteurised full cream bovine milk was used to prepare spiked samples for analytical evaluation. Purified *S. uberis* genomic DNA was extracted from an *S. uberis* isolate obtained from the Elizabeth Macarthur Agricultural Institute (EMAI), New South Wales, Australia, and confirmed by bacterial culture and MALDI TOF MS. DNA was extracted using the Wizard^®^ SV Genomic DNA Purification System (Promega Corporation, Australia). The DNA concentration was determined by spectrophotometric measurement using a NanoDrop spectrophotometer (Thermo Fisher Scientific, Melbourne, Australia).

To prepare the spiked milk samples, purified genomic DNA was added to pasteurised milk in a sterile tube to achieve a starting concentration of 100 ng/mL. A tenfold serial dilution series was then prepared by transferring an aliquot of the spiked milk into fresh pasteurised milk in subsequent sterile tubes. This generated a dilution series ranging from 1 × 10^2^ to 1 × 10^−8^ ng/mL across ten tubes. A negative-control tube containing pasteurised milk without added DNA was included. All dilutions were prepared and tested in triplicate.

DNA was extracted from 150 µL of each dilution using both the optimised HS method and the Wizard^®^ Genomic DNA Purification Kit (Promega Corporation, Australia). The Wizard kit yielded a final elution volume of 100 µL, while the HS method produced a final volume of 300 µL after neutralisation. Extracted DNA (2 µL) from each dilution was used as a template for LAMP and conventional PCR assays. Results were expressed as limit of detection (LOD) to enable comparison between the two DNA extraction methods.

### 2.7. Diagnostic Sensitivity Assessment Using Clinical Milk Samples

Following the analytical evaluation using spiked milk samples, the diagnostic sensitivity of the optimised HS method was assessed using clinical milk samples obtained from the Elizabeth Macarthur Agricultural Institute in Australia. A total of 514 clinical bovine milk samples submitted for mastitis investigation were screened by bacterial culture and matrix-assisted laser desorption ionisation time of flight mass spectrometry (MALDI TOF MS). Of these, 17 samples were confirmed positive for *S. uberis*. DNA was extracted from these 17 samples using the optimised HS method and tested using colorimetric LAMP and conventional PCR assays. Diagnostic sensitivity was calculated using a 2 × 2 contingency table, with bacterial culture as the reference method. Calculations were performed using the MedCalc online diagnostic test evaluation tool (https://www.medcalc.org/calc/diagnostic_test.php) (accessed on 25 December 2025).

Moreover, the McNemar’s test was used to compare detection outcomes of the LAMP and PCR assays conducted on DNA extracted using the HS method. The test assessed discordance between paired qualitative results from the same milk samples. Statistical significance was set at *p* < 0.05. Calculations were performed using the MedCalc online diagnostic test evaluation tool available at https://www.medcalc.org/en/calc/mcnemar.php (accessed on 30 January 2026).

### 2.8. Specificity Assessment

The specificity of the colorimetric LAMP and PCR assays was evaluated by testing the primers against a panel of non-target bacterial species selected to assess potential cross reactivity. The non-target bacterial isolates were obtained from the Veterinary Diagnostic Laboratory at Charles Sturt University, Wagga Wagga, Australia. The panel included *Pasteurella multocida*, *Erysipelothrix* spp., *Staphylococcus aureus*, *Escherichia coli*, *Mycoplasma synoviae*, *Mycoplasma gallisepticum*, *Streptococcus zooepidemicus*, *Pseudomonas aeruginosa*, *Enterococcus faecalis* and *Salmonella* Typhimurium. Genomic DNA from *S. uberis* was used as a positive control, and distilled water was included as a negative control. Genomic DNA for all non-target species was extracted using the Wizard^®^ Genomic DNA Purification Kit (Promega Corporation, Australia).

### 2.9. Clinical Specificity Assessment Using Non-S. uberis Milk Samples

To assess the diagnostic specificity of the assays under the proposed HS-based work-flow, an additional set of 14 clinical bovine milk samples that were culture-negative for S. uberis was tested. These samples were obtained from the same EMAI collection and included samples positive for other bacterial species (*Pseudomonas aeruginosa*, *Klebsiella pneumoniae*, *Streptococcus dysgalactiae* with *Trueperella pyogenes*, *Escherichia coli*, *Streptococcus haemolytica*, *Serratia marcescens*, *Staphylococcus aureus*, *Enterococcus* sp. and *Staphylococcus chromogenes*) as well as samples with no bacterial growth. DNA was extracted from all 14 samples using the optimised HS method and tested by both colorimetric LAMP and conventional PCR. The composition of the non-*S. uberis* clinical sample panel is detailed in Table 2.

### 2.10. PCR Amplicon Sequencing

Selected PCR amplicons were submitted for Sanger sequencing to confirm the identity of the amplified products. Sequencing was performed by the Australian Genome Research Facility Ltd. (AGRF) in Brisbane, Australia. Sequence identity was verified by comparison with reference sequences in the NCBI database.

## 3. Results

### 3.1. Development and Optimisation of Colorimetric LAMP and PCR Assays

Under the optimised conditions, both assays were initially evaluated using purified *S. uberis* genomic DNA extracted from the confirmed EMAI isolate with the Wizard^®^ SV Genomic DNA Purification System. The colorimetric LAMP assay produced a clear colour change from pink to yellow, while conventional PCR using SU F3 and SU B3 primers consistently generated the expected 178 bp amplicon. No colour change or PCR amplification was observed in negative control reactions containing nuclease-free water. These results confirmed that both assays were functional and suitable for subsequent analytical evaluation.

### 3.2. Limit of Detection of LAMP and PCR Using Purified DNA Diluted in Nuclease-Free Water

**Figure 2:**
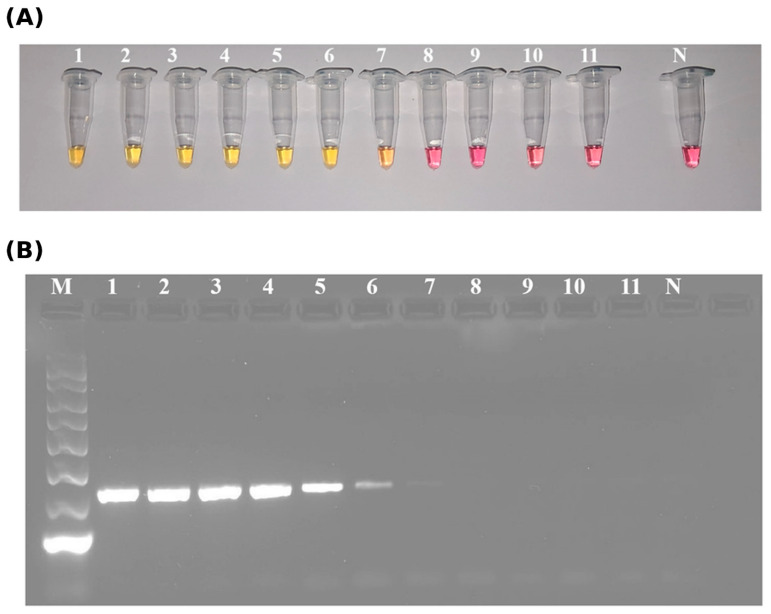
Analytical sensitivity of LAMP and PCR using serially diluted purified *S. uberis* genomic DNA. (**A**) Colorimetric LAMP results obtained from tenfold serial dilutions of purified DNA starting from 18.4 ng/µL. Samples 1 to 11 represent serial dilutions from 1.84 × 10^1^ to 1.84 × 10^−9^ ng/µL. A colour change from pink to yellow was interpreted as a positive result. (**B**) Agarose gel electrophoresis of PCR amplicons generated from the same serial dilutions. M indicates DNA ladder. N indicates negative control. The expected amplicon size was 178 bp.

The sensitivity of the LAMP and PCR assays was assessed using tenfold serial dilutions of purified *S. uberis* genomic DNA starting from a stock concentration of 18.4 ng/µL. LAMP consistently detected DNA at a concentration of 1.84 × 10^−4^ ng/µL (corresponding to approximately 92 genome copies per reaction), while PCR detected DNA at 1.84 × 10^−5^ ng/µL (approximately 9.2 genome copies per reaction). These results established the baseline LOD of each assay independent of the milk matrix. Representative colorimetric LAMP results and agarose gel electrophoresis of PCR amplicons from the purified DNA serial dilution are shown in Figure 2.

In the analytical milk experiment, because purified genomic DNA, rather than intact bacterial cells, was spiked into milk, the experiment evaluated assay performance in the presence of the milk matrix but did not assess bacterial lysis efficiency or DNA recovery during extraction.

### 3.3. Comparison of DNA Extraction Methods and Limit of Detection Using DNA-Spiked Pasteurised Bovine Milk

**Figure 3:**
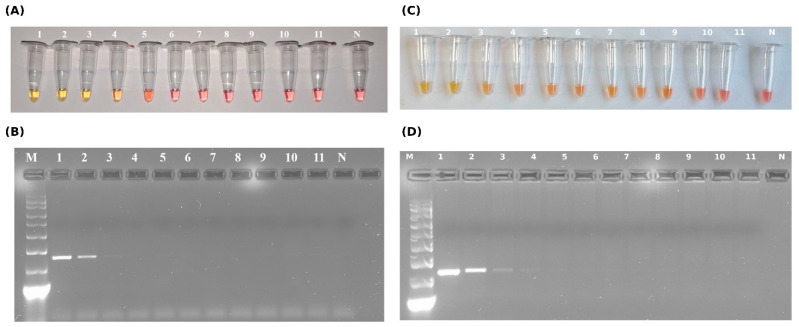
Determination of LOD for colorimetric LAMP and PCR using DNA-spiked pasteurised bovine milk. Colorimetric LAMP results (**A**,**C**) and agarose gel electrophoresis of PCR amplicons (**B**,**D**) using DNA extracted with the Wizard^®^ Genomic DNA Purification Kit (**A**,**B**) and the optimised HS method (**C**,**D**). Lanes 1 to 11 represent tenfold serial dilutions of spiked milk from 1 × 10^2^ to 1 × 10^−8^ ng/mL. N indicates negative control (non-spiked pasteurised milk). M indicates DNA ladder.

The limits of detection obtained for each DNA extraction method using colorimetric LAMP and conventional PCR are summarised in Table 3 and shown in Figure 3. Using the Wizard^®^ Genomic DNA Purification Kit, both LAMP and PCR detected *S. uberis* DNA at 1 × 10^0^ ng/mL, corresponding to approximately 5 × 10^5^ genome equivalents/mL and 1.5 × 10^3^ copies per reaction. This indicates equivalent performance of the two assays when DNA was extracted using the commercial kit. Using the optimised HS method, LAMP detected *S. uberis* DNA at a concentration of 1 × 10^1^ ng/mL (approximately 5 × 10^6^ genome equivalents/mL and approximately 5 × 10^3^ copies per reaction), while PCR achieved detection at 1 × 10^0^ ng/mL (approximately 5 × 10^5^ genome equivalents/mL and approximately 5 × 10^2^ copies per reaction). The HS method therefore showed a one log reduction in LAMP sensitivity compared to the Wizard kit, while PCR sensitivity remained equivalent between the two extraction methods. The difference in copies per reaction between the two methods at equivalent milk concentrations reflects the larger final volume of the HS method (300 µL) compared to the Wizard kit (100 µL), resulting in a threefold dilution of extracted DNA.

### 3.4. Clinical Validation and Diagnostic Sensitivity of the Colorimetric LAMP and PCR Assays

**Figure 4:**
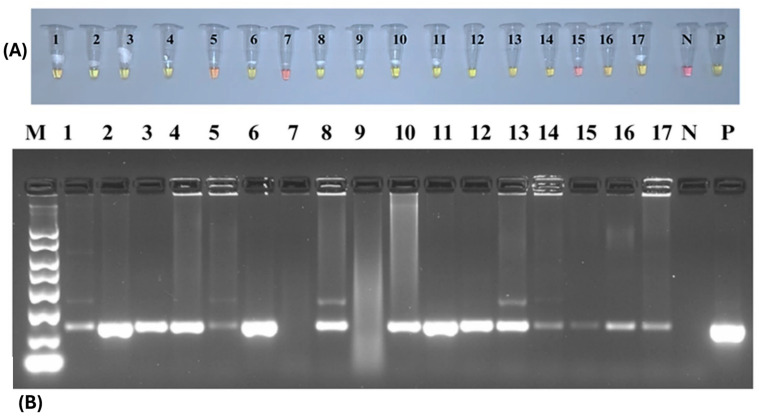
Clinical validation of the colorimetric LAMP and PCR assays for detection of *S. uberis* in bovine milk samples. (**A**) Colorimetric LAMP results; (**B**) agarose gel electrophoresis of PCR amplicons for 17 clinical milk samples (lanes 1 to 17) using template DNA extracted with the optimised HS method. M indicates DNA ladder, N indicates negative control, and P indicates positive control.

The diagnostic sensitivity of the colorimetric LAMP and PCR assays was assessed using 17 clinical bovine milk samples that were culture-positive for *S. uberis*. These samples were identified from a total of 514 clinical bovine milk samples submitted for mastitis investigation to the Elizabeth Macarthur Agricultural Institute. DNA was extracted from all 17 samples using the optimised HS method. Among the 17 clinical milk samples tested, 14 were positive by colorimetric LAMP, corresponding to a diagnostic sensitivity of 82.35% (95% CI: 0.56–0.96). A total of 15 samples were positive by conventional PCR, yielding a diagnostic sensitivity of 88.24% (95% CI: 0.64–0.99). No amplification was observed in negative-control samples (non-spiked pasteurised milk) for either assay. Representative colorimetric LAMP results and corresponding PCR amplification profiles for the clinical milk samples are shown in Figure 4.

### 3.5. Clinical Specificity Using Non-S. uberis Milk Samples

**Figure 5:**
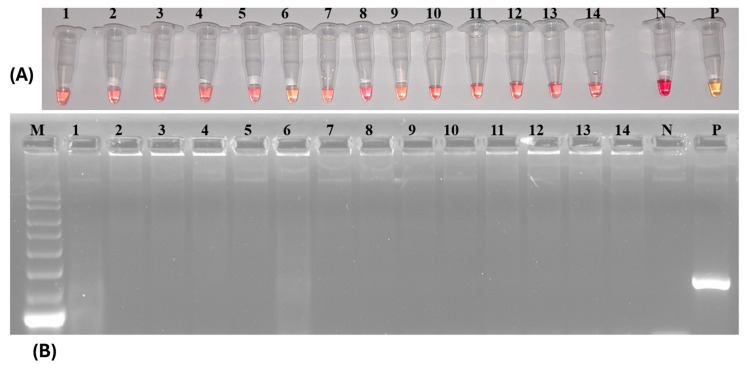
Validation of the colorimetric LAMP and PCR assays using culture-negative and non-*S. uberis* clinical milk samples. (**A**) Colorimetric LAMP results; (**B**) agarose gel electrophoresis of PCR amplicons for 14 culture-negative and non-*S. uberis* clinical milk samples (lanes 1 to 14, detailed in Table 2) using template DNA extracted with the optimised HS method. M indicates DNA ladder, N indicates negative control, and P indicates positive control.

Among the 14 clinical milk samples that were culture-negative for *S. uberis*, none produced a positive result by either colorimetric LAMP or conventional PCR (0/14; 100% diagnostic specificity). These samples included milk positive for nine different non-target bacterial species as well as four samples with no bacterial growth (Table 2 and Figure 5). DNA was extracted from all samples using the optimised HS method, confirming the specificity of both assays under the proposed field workflow. Combined with the 17 culture-positive samples, the full clinical evaluation comprised 31 clinical milk samples (17 *S. uberis*-positive and 14 *S. uberis*-negative), enabling calculation of both diagnostic sensitivity and specificity.

### 3.6. Specificity of the Colorimetric LAMP and PCR Assays

**Figure 6:**
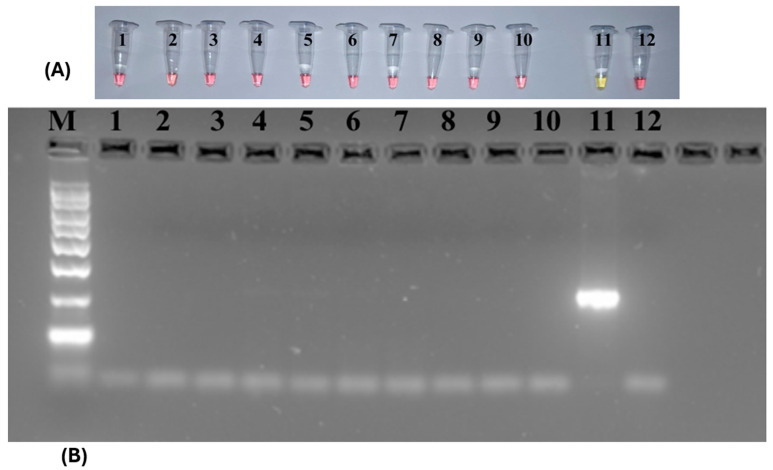
Specificity assessment of the colorimetric LAMP and PCR assays. (**A**) Colorimetric LAMP results; (**B**) agarose gel electrophoresis of PCR amplicons using primers designed for *S. uberis*. Lanes 1 to 10 correspond to non-target bacterial species: 1, *Pasteurella multocida*; 2, *Erysipelothrix* spp.; 3, *Staphylococcus aureus*; 4, *Escherichia coli*; 5, *Mycoplasma synoviae*; 6, *Mycoplasma gallisepticum*; 7, *Streptococcus zooepidemicus*; 8, *Pseudomonas aeruginosa*; 9, *Enterococcus faecalis*; 10, *Salmonella* Typhimurium. Lane 11 corresponds to *S. uberis* and lane 12 represents the negative control. M indicates DNA ladder.

The colorimetric LAMP and PCR assays showed no cross-reactivity with any of the 10 non-target bacterial species tested (Table 4 and Figure 6). Only *S. uberis* produced a positive result in both assays. Sequencing of selected PCR amplicons confirmed their identity as *S. uberis*, and the sequences were deposited in NCBI under accession numbers PV450694, PV459210 and PV459211. A summary of assay performance across the different extraction methods and assay formats is presented in Table 5.

## 4. Discussion

The optimised HS method produced DNA of sufficient quality for both LAMP and PCR detection of *S. uberis* in bovine milk. The LOD values obtained using HS-extracted DNA were moderately higher for LAMP but equivalent for PCR when compared with the Wizard^®^ Genomic DNA Purification Kit. These results demonstrate that a colour-based LAMP assay combined with a simplified DNA preparation method might provide a practical approach for detecting this pathogen. This finding supports the concept that complex and resource-intensive DNA extraction procedures are not always necessary for reliable molecular detection, particularly for decentralised or on-farm testing applications [38].

The LOD results summarised in Table 5 show that, when tested using DNA-spiked pasteurised bovine milk and the optimised HS method, the LAMP assay detected *S. uberis* DNA within one order of magnitude of PCR. Although PCR achieved a lower detection limit, both assays detected *S. uberis* DNA at relatively low concentrations, indicating that the HS method provided DNA suitable for reliable detection by either method. This finding aligns with previous studies reporting that LAMP is a robust amplification method capable of tolerating some inhibitors and operating effectively under isothermal conditions [39]. However, the results also confirm that LAMP performance remains dependent on DNA quality, reinforcing that DNA preparation is still a critical step even for isothermal amplification assays [40,41].

As summarised in Table 5, the HS method showed a one log higher LOD for LAMP than the Wizard^®^ Genomic DNA Purification Kit, while PCR showed the same LOD with both extraction methods. The Wizard kit is an established column-based extraction system. The HS method produces a larger final volume (300 µL) than the Wizard kit (100 µL), which results in a lower DNA concentration per unit volume extract. This is a known characteristic of the HS protocol design, where the combined lysis and neutralisation steps determine the final volume. Adjusting volumes to match the two methods was not performed, as the aim was to evaluate the HS workflow in its unmodified form as intended for field use. Despite this inherent dilution effect, the moderately reduced LAMP performance of the HS method relative to the commercial kit is notable given its minimal cost, simple reagent composition and short processing time of approximately 10 min. Future optimisation of the HS protocol could explore whether reducing the neutralisation volume improves detection limits without compromising workflow simplicity. This balance between analytical performance and operational simplicity supports the suitability of the HS method for on-farm diagnostic applications where access to laboratory equipment is limited [29,38].

The LAMP primers were designed to target the 16S rRNA gene of *S. uberis*. The 16S rRNA gene contains both conserved and variable regions. While the conserved regions are commonly used for broad classification of bacterial groups, the variable regions allow differentiation at the species level [42,43,44,45]. The region of the 16S rRNA gene targeted in this study enabled specific detection of *S. uberis* without cross-reactivity to the non-target bacterial species tested, including bacteria commonly associated with bovine mastitis or found in the milk environment, as well as *Streptococcus zooepidemicus*. However, this analytical specificity assessment was performed using DNA extracted with the commercial kit, and further testing using HS-extracted non-target bacteria in milk would be useful to confirm specificity under conditions that more closely reflect the proposed field workflow. High analytical specificity remains important for mastitis diagnostics because milk samples may contain diverse microbial populations. Accurate pathogen identification is critical for guiding appropriate treatment and avoiding unnecessary antimicrobial use [46,47,48].

An incubation time of 90 min was used for the LAMP assay when template DNA was prepared using the HS method. This was longer than the 30 to 45 min recommended by the manufacturer for purified DNA templates [49,50]. When purified DNA from the commercial kit was used, positive colour changes were typically observed within 60 min. The extended reaction time was necessary to allow sufficient amplification from crude DNA preparations, which may contain inhibitory substances such as milk fat and proteins [51]. Despite the longer incubation, the extended time did not adversely affect assay specificity or result in false-positive reactions. Unlike LAMP, conventional PCR achieved consistent amplification using HS-extracted DNA without requiring modification of the cycling conditions.

When applied to clinical milk samples, the LAMP assay detected *S. uberis* in 14 of 17 culture-positive samples, with a diagnostic sensitivity of 82.35%. PCR detected 15 of 17 samples, with a diagnostic sensitivity of 88.24%. The reduced sensitivity observed for LAMP in clinical samples may be attributed to lower bacterial loads, heterogeneous distribution of bacteria in milk or the presence of inhibitory components that affect colour development or amplification efficiency [41,51]. These factors are well recognised challenges in direct milk testing and highlight the trade-off between assay simplicity and detection performance in field deployable diagnostics [51,52]. The diagnostic sensitivity observed for LAMP was lower than 96% reported by Cornelissen, De Greeff, Heuvelink, Swarts, Smith, Van der Wal and Health4Food-Dutch Mastitis Diagnostics [36] for a non-colorimetric LAMP assay targeting *S. uberis*. However, that study utilised a laboratory-based DNA extraction kit, whereas the clinical sample evaluation in the present study used the simplified HS method only. Therefore, direct comparison between the two studies should be made cautiously, and future work should assess paired HS and commercial kit extraction on the same clinical milk samples. Several rapid DNA extraction methods have been reported for use with milk samples in mastitis diagnostics [52,53,54]. These methods are generally based on chemical, enzymatic or combined approaches. Although many are faster than conventional protocols, most still rely on laboratory equipment such as centrifuges, which limits their use under field conditions [55]. The HS method used in this study requires only basic heating equipment and two reagent solutions. It can be completed in approximately 10 min. As a crude extraction method, some background smearing was observed on agarose gel images. This is likely due to co extraction of proteins, RNA and somatic cell DNA from the milk matrix [56,57]. However, this did not significantly affect assay performance.

Despite the promising results obtained in this study, several limitations should be acknowledged. The number of clinical milk samples used for assay validation was relatively small. This limits the precision of the estimated diagnostic sensitivity and may not fully capture the variability present under field conditions. Clinical samples were pre-characterised as *S. uberis*-positive by bacterial culture. Inclusion of a larger and more diverse sample set, including culture-negative samples and samples with mixed infections, would allow more comprehensive evaluation of diagnostic specificity. The analytical evaluation was performed using purified DNA spiked into pasteurised milk rather than live bacterial cells. Therefore, bacterial lysis efficiency and DNA recovery were not assessed in this particular experiment. This does not fully replicate the DNA extraction dynamics encountered with intact bacterial cells in clinical samples. Future validation studies should include spiking with live *S. uberis* isolates to express LOD as CFU/mL, which would improve comparability with published studies and better reflect field conditions. The specificity panel did not include *Streptococcus parauberis*, which is genetically closely related to *S. uberis*, and future studies should include this and other closely related streptococcal species. Furthermore, although the colour-based readout simplifies result interpretation, borderline colour changes may introduce subjectivity, particularly at low DNA concentrations. A limitation of this study is the use of a small number of technical replicates (*n* = 3), which restricts robust statistical estimation of LOD using probability-based models. Future work should focus on larger-scale clinical validation and evaluation under true field conditions to further assess assay robustness, usability and impact on mastitis management practices.

The HS method of DNA extraction combined with LAMP or PCR shows potential for rapid, sensitive and low-cost on-farm diagnosis of bovine mastitis, particularly in re-source-limited settings. Future studies should validate the method using larger-field sam-ple sets and multiple mastitis pathogens. The current assays provide qualitative detection and have not been evaluated for quantifying infection intensity. Future research should therefore explore quantitative molecular approaches, such as real-time PCR or quantitative LAMP, to measure bacterial load and its association with disease severity.

In conclusion, this study demonstrated that combining a colour-based LAMP assay with the HS DNA preparation method provided a balanced solution between analytical performance and operational simplicity. While PCR remains slightly more sensitive, the LAMP HS workflow reduces reliance on laboratory infrastructure, specialised equipment and trained personnel. If validated further under field conditions, the ability to detect *S. uberis* directly from milk using a simplified workflow has practical implications for mastitis management. Early identification of the causative pathogen enables targeted treatment decisions and reduces reliance on broad-spectrum antimicrobials. This is particularly relevant for *S. uberis*, which requires management strategies that may differ from those applied to contagious mastitis pathogens.

## Figures and Tables

**Table 1:** Sequences and positions of primers targeting the 16S rRNA gene of *S. uberis* used for LAMP and PCR.

Primer	Primer Sequences 5′-3′	Positions of Primers
SU-F3	AAAAGGGGCAAATGCTTCAC	181–200
SU-B3	GGAATCTTCGGCAATGGGG	340–358
SU-FIP	CGTCGCCTTGGTAAGCCGTTTATGAGATGGACCTGCGTTG	241–260 and 201–220
SU-BIP	ACATAGCCGACCTGAGAGGGTTACTGCTGCCTCCCGTAG	263–283 and 321–338
SU-LB	TCGGCCACACTGGGACT	286–302

Positions of primers are given relative to the 16S rRNA gene sequence of *S. uberis* (GenBank accession AB023576.1). Inner primers FIP and BIP are composite primers derived from internal regions of the target gene.

**Table 2:** Culture-negative and non-*S. uberis* clinical milk samples used to validate the colorimetric LAMP and conventional PCR assays.

Sample No.	Milk Culture Results and Samples	Growth Notes	Result	
			LAMP	PCR
1.	*Pseudomonas aeruginosa*	Pure growth	−	−
2.	*Klebsiella pneuomoniae*	Pure growth	−	−
3.	*Streptococcus dysgalactiae* and *Trueperella pyogenes*	Mixed growth	−	−
4.	No growth	NA	−	−
5.	*Escherichia coli*	Pure growth	−	−
6.	*Klebsiella pneuomoniae*	Pure growth	−	−
7.	No growth	NA	−	−
8.	*Streptococcus haemolytica*	Pure growth	−	−
9.	*Serratia marcescens*	Enrichment	−	−
10.	No growth	NA	−	−
11.	*Staphylococcus aureus*	Pure growth	−	−
12.	No growth	NA	−	−
13.	*Enterococcus* sp.	Pure growth	−	−
14.	*Staphylococcus chromogenes*	Pure growth	−	−
15.	Negative control, nuclease-free water	NA	−	−
16.	Positive control, *Streptococcus uberis*	Pure growth	+	+

NA—not applicable, + positive result. − negative result.

**Table 3:** Limit of detection of *S. uberis* determined by LAMP and PCR using DNA-spiked pasteurised bovine milk with two DNA extraction methods.

Well Number	Dilution	Conc. (ng/mL)	Genome eq./mL	Copies per Reaction		Wizard^®^ Kit		HS Method	
				Wizard^®^	HS	LAMP	PCR	LAMP	PCR
1	10^0^	1 × 10^2^	~5 × 10^7^	~1.5 × 10^5^	~5 × 10^4^	+	+	+	+
2	10^−1^	1 × 10^1^	~5 × 10^6^	~1.5 × 10^4^	~5 × 10^3^	+	+	+ *	+
3	10^−2^	1 × 10^0^	~5 × 10^5^	~1.5 × 10^3^	~5 × 10^2^	+ *	+ *	−	+ *
4	10^−3^	1 × 10^−1^	~5 × 10^4^	~1.5 × 10^2^	~5 × 10^1^	−	−	−	−
5	10^−4^	1 × 10^−2^	~5 × 10^3^	~1.5 × 10^1^	~5 × 10^0^	−	−	−	−
6	10^−5^	1 × 10^−3^	~5 × 10^2^	~1.5 × 10^0^	~5 × 10^−1^	−	−	−	−
7	10^−6^	1 × 10^−4^	~5 × 10^1^	~1.5 × 10^−1^	~5 × 10^−2^	−	−	−	−
8	10^−7^	1 × 10^−5^	~5 × 10^0^	~1.5 × 10^−2^	~5 × 10^−3^	−	−	−	−
9	10^−8^	1 × 10^−6^	~5 × 10^−1^	~1.5 × 10^−3^	~5 × 10^−4^	−	−	−	−
10	10^−9^	1 × 10^−7^	~5 × 10^−2^	~1.5 × 10^−4^	~5 × 10^−5^	−	−	−	−
11	10^−10^	1 × 10^−8^	~5 × 10^−3^	~1.5 × 10^−5^	~5 × 10^−6^	−	−	−	−
N	Milk only	−	−	−	−	−	−	−	−

+ indicates a positive result in all three replicates; − indicates a negative result in all three replicates. * LOD: the lowest DNA concentration at which all three replicates were positive. HS: optimised HotSHOT method; Wizard^®^ Kit: Wizard^®^ Genomic DNA Purification Kit (Promega Corporation, Australia). Genome equivalents were estimated based on the *S. uberis* genome size of approximately 1.8 Mb (~2 fg per genome copy). Copies per reaction were calculated based on the volume of milk used for extraction (150 µL), the elution volume (100 µL for the Wizard^®^ Kit; 300 µL for the HS method) and the template volume added to each reaction (2 µL). For the Wizard^®^ Kit, each reaction contained DNA equivalent to 3 µL of the original milk sample. For the HS method, each reaction contained DNA equivalent to 1 µL of the original milk sample.

**Table 4:** Analytical specificity of LAMP and PCR primers tested against non-target bacterial species.

Bacterial Species Tested	LAMP Result	PCR Result
*Pasteurella multocida*	−	−
*Erysipelothrix* spp.	−	−
*Staphylococcus aureus*	−	−
*Escherichia coli*	−	−
*Mycoplasma synoviae*	−	−
*Mycoplasma gallisepticum*	−	−
*Streptococcus zooepidemicus*	−	−
*Pseudomonas aeruginosa*	−	−
*Enterococcus faecalis*	−	−
*Salmonella typhimurium*	−	−
*Streptococcus uberis*	+	+
Negative control	−	−

+, positive; −, negative.

**Table 5:** Summary of assay performance using different DNA extraction methods and molecular assays.

Evaluation Parameter	LAMP HS	LAMP Wizard	PCR HS	PCR Wizard
LOD using purified DNA in nuclease-free water *	1.84 × 10^−4^ ng/µL	NP	1.84 × 10^−5^ ng/µL	NP
LOD using purified isolate in spiked pasteurised milk sample	1 × 10^1^ ng/mL	1 × 10^0^ ng/mL	1 × 10^0^ ng/mL	1 × 10^0^ ng/mL
Diagnostic sensitivity using culture-positive clinical bovine mastitis samples	82.35% (14/17)	NP	88.24% (15/17)	NP
Analytical specificity using non-target isolates	NP	100% (10/10)	NP	100% (10/10)

* Extraction not required because purified genomic DNA diluted in nuclease-free water was used directly as template. NP—not performed.

## Data Availability

The data supporting the findings of this study are available from the corresponding author upon reasonable request. In addition, the relevant datasets have been deposited in the NCBI database under the accession numbers listed in the Section 3 and also the related article we published (https://doi.org/10.1016/j.tvjl.2026.106583).

## References

[1] Ghumman N.Z., Bruce M., Barbosa A.D., Ijaz M., Peng J., Gogoi-Tiwari J. (2026). Bovine mastitis and antimicrobial resistance in Pakistan’s dairy sector: Current status and future prospects. Vet. Res. Commun..

[2] Hogeveen H., Pyorala S., Waller K.P., Hogan J.S., Lam T., Oliver S.P., Schukken Y.H., Barkema H.W., Hillerton J.E. Current status and future challenges in mastitis research. Proceedings of the 50th Annual Meeting of the National Mastitis Council, 2011.

[3] Ramuada M., Tyasi T.L., Gumede L., Chitura T. (2024). A practical guide to diagnosing bovine mastitis: A review. Front. Anim. Sci..

[4] Tomanic D., Kladar N., Kovacevic Z. (2025). Antibiotic Residues in Milk as a Consequence of Mastitis Treatment: Balancing Animal Welfare and One Health Risks. Vet. Sci..

[5] Servellon F.J.G., Renaud D.L., Osorio B.J.P., Spence K.L., DeVries T.J., Serrenho R.C. (2025). Validation of a rapid on-farm culture system for group classification of clinical mastitis-causing pathogens. JDS Commun..

[6] Preine F., Herrera D., Scherpenzeel C., Kalmus P., McCoy F., Smulski S., Rajala-Schultz P., Schmenger A., Moroni P., Kromker V. (2022). Different European Perspectives on the Treatment of Clinical Mastitis in Lactation. Antibiotics.

[7] Wilm J., Svennesen L., Ostergaard Eriksen E., Halasa T., Kromker V. (2021). Veterinary Treatment Approach and Antibiotic Usage for Clinical Mastitis in Danish Dairy Herds. Antibiotics.

[8] Ferronatto J.A., Ferronatto T.C., Schneider M., Pessoa L.F., Blagitz M.G., Heinemann M.B., Della Libera A.M.M.P., Souza F.N. (2018). Diagnosing mastitis in early lactation: Use of Somaticell^®^, California mastitis test and somatic cell count. Ital. J. Anim. Sci..

[9] Peckler G.L., Adcock S.J.J. (2025). Evaluating somatic cell count, the California mastitis test, and infrared thermography for subclinical mastitis detection in meat ewes. Res. Vet. Sci..

[10] Ajose D.J., Oluwarinde B.O., Abolarinwa T.O., Fri J., Montso K.P., Fayemi O.E., Aremu A.O., Ateba C.N. (2022). Combating Bovine Mastitis in the Dairy Sector in an Era of Antimicrobial Resistance: Ethno-veterinary Medicinal Option as a Viable Alternative Approach. Front. Vet. Sci..

[11] Saeed S.I., Kamaruzzaman N.F., Gahamanyi N., Nguyen T.T.H., Hossain D., Kahwa I. (2024). Correction: Confronting the complexities of antimicrobial management for *Staphylococcus aureus* causing bovine mastitis: An innovative paradigm. Ir. Vet. J..

[12] Duarte C.M., Freitas P.P., Bexiga R. (2015). Technological advances in bovine mastitis diagnosis: An overview. J. Vet. Diagn. Investig..

[13] Kajdanek A., Kluska M., Matusiak R., Kazimierczak J., Dastych J. (2024). A Rapid and Inexpensive PCR Test for Mastitis Diagnosis Based on NGS Data. Pathogens.

[14] Koskinen M.T., Holopainen J., Pyorala S., Bredbacka P., Pitkala A., Barkema H.W., Bexiga R., Roberson J., Solverod L., Piccinini R. (2009). Analytical specificity and sensitivity of a real-time polymerase chain reaction assay for identification of bovine mastitis pathogens. J. Dairy Sci..

[15] Hiitio H., Riva R., Autio T., Pohjanvirta T., Holopainen J., Pyorala S., Pelkonen S. (2015). Performance of a real-time PCR assay in routine bovine mastitis diagnostics compared with in-depth conventional culture. J. Dairy Res..

[16] Riffon R., Sayasith K., Khalil H., Dubreuil P., Drolet M., Lagacé J. (2001). Development of a rapid and sensitive test for identification of major pathogens in bovine mastitis by PCR. J. Clin. Microbiol..

[17] Shome B.R., Das Mitra S., Bhuvana M., Krithiga N., Velu D., Shome R., Isloor S., Barbuddhe S.B., Rahman H. (2011). Multiplex PCR assay for species identification of bovine mastitis pathogens. J. Appl. Microbiol..

[18] Syring C., Boss R., Reist M., Bodmer M., Hummerjohann J., Gehrig P., Graber H.U. (2012). Bovine mastitis: The diagnostic properties of a PCR-based assay to monitor the *Staphylococcus aureus* genotype B status of a herd, using bulk tank milk. J. Dairy Sci..

[19] Garner C., Stephen C., Pant S.D., Ghorashi S.A. (2023). Comparison of PCR-HRM, colorimetric LAMP and culture based diagnostic assays in the detection of endometritis caused by *Streptococcus equi* subsp. *zooepidemicus* in mares. Vet. Res. Commun..

[20] Ghorashi M.S., Pant S.D., Ghorashi S.A. (2022). Comparison of colourimetric loop-mediated isothermal amplification (LAMP), PCR and high-resolution melt curve analysis and culture-based diagnostic assays in the detection of three salmonella serotypes in poultry. Avian Pathol..

[21] Poussard M., Pant S.D., Huang J., Scott P., Ghorashi S.A. (2025). Comparative evaluation of PCR and loop-mediated isothermal amplification (LAMP) assays for detecting *Pasteurella multocida* in poultry. N. Z. Vet. J..

[22] Wong S.A., Woodgate R.G., Pant S.D., Ghorashi S.A. (2020). Rapid detection of *Bovicola ovis* using colourimetric loop-mediated isothermal amplification (LAMP): A potential tool for the detection of sheep lice infestation on farm. Parasitol. Res..

[23] Notomi T., Okayama H., Masubuchi H., Yonekawa T., Watanabe K., Amino N., Hase T. (2000). Loop-mediated isothermal amplification of DNA. Nucleic Acids Res..

[24] Cui S., Wei Y., Li C., Zhang J., Zhao Y., Peng X., Sun F. (2024). Visual Loop-Mediated Isothermal Amplification (LAMP) Assay for Rapid On-Site Detection of *Escherichia coli O157*: *H7* in Milk Products. Foods.

[25] Tanner N.A., Zhang Y., Evans T.C. (2015). Visual detection of isothermal nucleic acid amplification using pH-sensitive dyes. Biotechniques.

[26] Kapalamula T.F., Thapa J., Akapelwa M.L., Hayashida K., Gordon S.V., Hang’ Ombe B.M., Munyeme M., Solo E.S., Bwalya P., Nyenje M.E. (2021). Development of a loop-mediated isothermal amplification (LAMP) method for specific detection of *Mycobacterium bovis*. PLoS Neglected Trop. Dis..

[27] Pascual-Garrigos A., Maruthamuthu M.K., Ault A., Davidson J.L., Rudakov G., Pillai D., Koziol J., Schoonmaker J.P., Johnson T., Verma M.S. (2021). On-farm colorimetric detection of *Pasteurella multocida*, *Mannheimia haemolytica*, and *Histophilus somni* in crude bovine nasal samples. Vet. Res..

[28] Ghorashi S.A., Bradbury J.M., Ferguson-Noel N.M., Noormohammadi A.H. (2013). Comparison of multiple genes and 16S-23S rRNA intergenic space region for their capacity in high resolution melt curve analysis to differentiate *Mycoplasma gallisepticum* vaccine strain ts-11 from field strains. Vet. Microbiol..

[29] Jember T.F., Westman M.E., Pant S.D., Ghorashi S.A. (2026). Field-deployable molecular workflow for detection, resistance screening, and genotyping of *Staphylococcus aureus* in bovine mastitis. Vet. J..

[30] Ravine D., Suthers G. (2012). Quality standards and samples in genetic testing. J. Clin. Pathol..

[31] Paul R., Ostermann E., Wei Q. (2020). Advances in point-of-care nucleic acid extraction technologies for rapid diagnosis of human and plant diseases. Biosens. Bioelectron..

[32] Date Chong M., Sheehan S., Battaglia J., Wescott D.J., Wallin J. (2023). Comparative study of Rapid DNA versus conventional methods on compromised bones. Forensic Sci. Int. Genet..

[33] Silva R.C.D., de Lima S.C., Dos Santos Reis W.P.M., de Magalhaes J.J.F., Magalhaes R.N.O., Rathi B., Kohl A., Bezerra M.A.C., Pena L. (2023). Comparison of DNA extraction methods for COVID-19 host genetics studies. PLoS ONE.

[34] Zou Y., Mason M.G., Wang Y., Wee E., Turni C., Blackall P.J., Trau M., Botella J.R. (2017). Nucleic acid purification from plants, animals and microbes in under 30 seconds. PLoS Biol..

[35] Goulart D.B., Mellata M. (2022). *Escherichia coli* Mastitis in Dairy Cattle: Etiology, Diagnosis, and Treatment Challenges. Front. Microbiol..

[36] Cornelissen J., De Greeff A., Heuvelink A.E., Swarts M., Smith H.E., Van der Wal F.J., Health4Food-Dutch Mastitis Diagnostics C. (2016). Rapid detection of *Streptococcus uberis* in raw milk by loop-mediated isothermal amplification. J. Dairy Sci..

[37] Griffioen K., Cornelissen J., Heuvelink A., Adusei D., Mevius D., Jan van der Wal F., Health4Food-Dutch Mastitis Diagnostics C. (2020). Development and evaluation of 4 loop-mediated isothermal amplification assays to detect mastitis-causing bacteria in bovine milk samples. J. Dairy Sci..

[38] Shwani A., Zuo B., Alrubaye A., Zhao J., Rhoads D.D. (2023). A simple, inexpensive alkaline method for bacterial DNA extraction from environmental samples for PCR surveillance and microbiome analyses. Appl. Sci..

[39] Jackson K.R., Layne T., Dent D.A., Tsuei A., Li J., Haverstick D.M., Landers J.P. (2020). A novel loop-mediated isothermal amplification method for identification of four body fluids with smartphone detection. Forensic Sci. Int. Genet..

[40] Moehling T.J., Choi G., Dugan L.C., Salit M., Meagher R.J. (2021). LAMP Diagnostics at the Point-of-Care: Emerging Trends and Perspectives for the Developer Community. Expert Rev. Mol. Diagn..

[41] Nwe M.K., Jangpromma N., Taemaitree L. (2024). Evaluation of molecular inhibitors of loop-mediated isothermal amplification (LAMP). Sci. Rep..

[42] Bartos O., Chmel M., Swierczková I. (2024). The overlooked evolutionary dynamics of 16S rRNA revises its role as the “gold standard” for bacterial species identification. Sci. Rep..

[43] Clarridge J.E. (2004). Impact of 16S rRNA gene sequence analysis for identification of bacteria on clinical microbiology and infectious diseases. Clin. Microbiol. Rev..

[44] Johnson J.S., Spakowicz D.J., Hong B.Y., Petersen L.M., Demkowicz P., Chen L., Leopold S.R., Hanson B.M., Agresta H.O., Gerstein M. (2019). Evaluation of 16S rRNA gene sequencing for species and strain-level microbiome analysis. Nat. Commun..

[45] Větrovský T., Baldrian P. (2013). The variability of the 16S rRNA gene in bacterial genomes and its consequences for bacterial community analyses. PLoS ONE.

[46] Bhattacharya S. (2013). Early diagnosis of resistant pathogens: How can it improve antimicrobial treatment?. Virulence.

[47] Ku T.S.N., Al Mohajer M., Newton J.A., Wilson M.H., Monsees E., Hayden M.K., Messacar K., Kisgen J.J., Diekema D.J., Morgan D.J. (2023). Improving antimicrobial use through better diagnosis: The relationship between diagnostic stewardship and antimicrobial stewardship. Infect. Control Hosp. Epidemiol..

[48] Yamin D., Uskokovic V., Wakil A.M., Goni M.D., Shamsuddin S.H., Mustafa F.H., Alfouzan W.A., Alissa M., Alshengeti A., Almaghrabi R.H. (2023). Current and Future Technologies for the Detection of Antibiotic-Resistant Bacteria. Diagnostics.

[49] Daskou M., Tsakogiannis D., Dimitriou T.G., Amoutzias G.D., Mossialos D., Kottaridi C., Gartzonika C., Markoulatos P. (2019). WarmStart colorimetric LAMP for the specific and rapid detection of HPV16 and HPV18 DNA. J. Virol. Methods.

[50] Patton G.C., Alpaslan E., Ren G., Zhang Y., Tanner N.A., Nichols N.M. Optimizing a rapid, isothermal workflow for detection of SARS-CoV-2 viral RNA using WarmStart^®^ LAMP Reagents with UDG. *New England Biolabs Application Note*, 2021, 1–5. https://www.neb.com/en/-/media/nebus/files/application-notes/appnote_optimizing_rapid_isothermal-workflow_for_sars-cov-2_with_warmstart_lamp_with_udg.pdf?rev=7cb6e927ebf54ac5bbb7851074562ca9&hash=AC132B9B39C2EABE1974090E5E6152A7&srsltid=AfmBOooZT4-W2Emkj98L_cYBilUq7xVAEnfKF21lptT4CbNNN6QoYc2K.

[51] Kase J.A., Pfefer T.L. (2016). Nucleic acid sample preparations from dairy products and milk. Sample Preparation Techniques for Soil, Plant, and Animal Samples.

[52] Cremonesi P., Castiglioni B., Malferrari G., Biunno I., Vimercati C., Moroni P., Morandi S., Luzzana M. (2006). Technical note: Improved method for rapid DNA extraction of mastitis pathogens directly from milk. J. Dairy Sci..

[53] Burbano E., Sierra S., Torres K., Mercado M., Carrascal A., Poutou R. (2006). Rapid DNA extraction and PCR validation for direct detection of Listeria monocytogenes in raw milk. Rev. MVZ Córdoba.

[54] Pancza B., Szathmáry M., Gyurján I., Bánkuti B., Tudós Z., Szathmary S., Stipkovits L., Sipos-Kozma Z., Asványi B., Varga L. (2021). A rapid and efficient DNA isolation method for qPCR-based detection of pathogenic and spoilage bacteria in milk. Food Control.

[55] Ali N., Rampazzo R.C.P., Costa A.D.T., Krieger M.A. (2017). Current Nucleic Acid Extraction Methods and Their Implications to Point-of-Care Diagnostics. Biomed. Res. Int..

[56] Tan T.T., Tan Z.Y., Tan W.L., Lee P.F. (2007). Gel electrophoresis: DNA Science Without the DNA!. Biochem. Mol. Biol. Educ..

[57] Westermeier R., Janson J.C. (2011). Electrophoresis in Gels.

